# Editorial: Chronic pain in neuropsychiatric diseases

**DOI:** 10.3389/fnhum.2023.1213217

**Published:** 2023-06-12

**Authors:** Daniela Adamo, Michele D. Mignogna, Xue-Qiang Wang, Grazia Daniela Femminella

**Affiliations:** ^1^Department of Neuroscience, Reproductive Sciences and Dentistry, University of Naples Federico II, Naples, Italy; ^2^Department of Sport Rehabilitation, University of Sport, Shanghai University, Shanghai, China; ^3^Department of Translational Medical Sciences, University of Naples Federico II, Naples, Italy

**Keywords:** neuropsychiatric disease, chronic pain, cognitive impairment, elderly, white matter changes

Chronic pain, mental health disorders, cognitive impairment are common and often overlap in the general population across the lifespan (Hooten, 2016; Harris, 2023), especially in the elderly that generally suffer from multi-morbidity (Lu et al., 2021). Epidemiological and review studies have proposed a complex interplay of chronic pain, mood disorders and dementia where physiological, emotional, cognitive, social, environmental factors and psychological factors may act synergically (Innes and Sambamoorthi, 2020; Tanaka and Vécsei, 2021). Bidirectional relationships between these conditions have been suggested, due to the fact that chronic pain further increases risk for psychiatric vulnerability or cognitive decline of affected patients (Bondesson et al., 2018; Innes and Sambamoorthi, 2020) moreover, mood disorders and dementia can make a person more sensitive to pain or aggravate pain, thus increasing socioeconomic costs and aggravating burden on caregivers and health care services (Kaufmann et al., 2021).

Growing evidence suggests that psychiatric disorders and cognitive impairment may be secondary to chronic pain (Zhang et al., 2021), while in other cases they precede the onset of pain or reflect alternative expressions of the same underlying psychobiological disorder (Velly and Mohit, 2018).

Irrespective of the initial trigger, the prevalence of anxiety and depression is around of 35–45% in several chronic and disabling pain conditions such as fibromyalgia, low back pain, irritable bowel syndrome, migraines, temporomandibular disorders, and burning mouth syndrome (Meda et al., 2022; Adamo et al., 2023) where mood disorders may not only contribute to pain intensity, but also predict the onset of dementia 25 years in advance with a consequent increased risk of disability for the patients (Ly et al., 2021).

Moreover, it's known that chronic pain may accelerate neurodegeneration in Alzheimer's disease and other types of dementia with an increased risk to develop these conditions up to 36% (Cao et al., 2019).

Specifically, people living with chronic pain at multiple sites of the body also have a faster rate of cognitive decline including a deficit of attention, memory, executive planning, and information processing especially in older age (Zhang et al., 2021). Old age is an important risk factor to develop dementia and chronic pain; therefore, in the elderly both conditions are generally presented simultaneously, nevertheless clinical recognition of patients' pain may be flawed by difficulties to report verbally, especially in advance cases of dementia. Thus, chronic pain is under recognized and not treated adequately in advanced cases even though the pain relief may also reduce the onset of neuropsychiatric symptoms such as agitation and aggression (Rouch et al., 2022).

Studies on functional imaging of the brain suggest that mental health disorders and chronic pain share biological mechanisms, which contribute to the interconnection, since the somatosensory cortex interacts with the amygdala, the hypothalamus and the anterior cingulate gyrus resulting in the mental and physical experience of pain (Martucci and Mackey, 2018). The same regions also contribute to anxiety, depression and memory dysfunction (De Ridder et al., 2021). Indeed, patients with chronic pain show a disruption of the default mode network and other brain networks, changes in brain morphology such as gray matter volume reduction in these brain regions, hippocampal atrophy, locus coeruleus dysfunction, and white matter changes that may be involved not only in pain processing and emotional regulation, but also in attention, memory consolidation, and cognitive processing (Cao et al., 2019; van Ettinger-Veenstra et al., 2019).

Recently, data about brain functionality, especially considering white matter integrity suggested that high prevalence of white matter hyperintensities (WMHs) in spinothalamic tract may have a role in the onset and/or in aggravation of mood disorders, cognitive decline and pain although it's not completely clear if chronic pain increase the risk of WMHs development or the early onset of WMHs may contribute later in life in the development of chronic pain (Calabria et al., 2023).

Untreated pain or high pain intensity may cause the progression of WMHs, accelerating brain aging that in turn may also aggravate mood disorders and cognitive decline (Adamo et al., 2022).

Moreover, it's essential to consider that pain intensity in chronic pain conditions has been related to established individual risk factors such as older age, female sex, lower level of education, higher BMI, reduced cognitive function, and higher levels of depression (Mills et al., 2019).

Multiple comorbidities, cardiovascular risk factors and uncorrected lifestyle behaviors (e.g., smoking, physical inactivity, and mid-life obesity) contributing to premature aging of the brain may not only aggravate chronic pain conditions but also mental and cognitive disorders. Specifically, hypertension, hypercholesterolemia, and hyperhomocisteinemia may increase the risk of developing small vessel disease, increasing cerebral hypoperfusion and exacerbating any preexisting conditions (Seib et al., 2022). Regarding plasma homocysteine level, clinical studies found that hyperhomocysteinemia is associated not only with an increased prevalence of mood disorders and more severe cognitive impairment but may also increase the risk of development of chronic neuropathic pain conditions through the up-regulation of the pronociceptive T-type calcium channels (Gaifullina et al., 2019). In addition, recent research suggest that healthy diet may protect cognitive function by attenuating the negative effects of changes in connectivity over time and improving chronic pain (Gaynor et al.).

Chronic pain's economic impact costs $635 billion annually in direct medical costs, loss of productivity, and disability programs (Gaskin and Richard, 2012); therefore, the management of these patients represents a challenge especially in the elderly with mood and cognitive disorders requiring the necessity of multidisciplinary centers that may address the multitude of factors that contribute to an individual's pain experience (Khera and Rangasamy, 2021).

An early treatment with specific drugs that work simultaneously on pain, mood and cognition is recommended to improve quality of life and reduce disability in these patients.

Recently vortioxetine, a multimodal antidepressant has demonstrated its efficacy in pain relief, anxiety, depression and sleep disturbances, improving cognition and neuroplasticity of the brain. In addition, this drug showed a good tolerability in several clinical studies; no drug interactions or side effects, such as QTc prolongation, sexual dysfunction, or weight gain have been generally reported.

Moreover, the treatment of insomnia is essential in patients with chronic pain especially using dual orexin receptor antagonists that have demonstrated high efficacy in inducing and maintaining sleep without impairing cognition (Adamo et al., 2021; Zhou et al., 2023).

Moreover, the early identification and monitoring of modifiable metabolic and vascular factors such as hypertension, cholesterol, and homocysteine level, a decrease in alcohol use and stopping smoking, the reduction of obesity promoting good nutrition, physical and cognitive activity may be essential to the successful rehabilitation of these complex patients (Seib et al., 2022).

Moreover, the integration of alternative strategies, including music interventions, into the multidisciplinary approach to chronic pain management has the potential to enhance patient wellbeing, reduce reliance on pharmacological interventions, and improve overall quality of life (Du et al.). As the field continues to evolve, further studies and collaborations are needed to explore and optimize the use of alternative strategies in the comprehensive care of individuals with chronic pain.

## Author contributions

All authors listed have made a substantial, direct, and intellectual contribution to the work and approved it for publication.
